# Bioinformatics Analysis Reveals PPR Genes Modulation by Ahyp-miR0005 Under Abiotic Stress Across Diverse Plant Species

**DOI:** 10.3390/plants14172757

**Published:** 2025-09-03

**Authors:** Vladimir Flores Benavides, Ricardo A. Chávez Montes, Flor de Fátima Rosas Cárdenas

**Affiliations:** 1 Centro de Investigación en Biotecnología Aplicada, Instituto Politécnico Nacional, Ex-Hacienda San Juan Molino Carretera Estatal Tecuexcomac-Tepetitla km 1.5, Tlaxcala 90700, Mexico; vfloresb1800@alumno.ipn.mx; 2 Department of Biology, Indiana University, Bloomington, IN 47405, USA; vonklatka@gmail.com

**Keywords:** PPR proteins, abiotic stress, amaranth, miRNAs, miRNA sponge

## Abstract

MicroRNAs (miRNAs) play a critical role in gene regulation in plants. Several members of the pentatricopeptide repeat (PPR) family have been identified as predicted targets of Ahyp-miR0005, a miRNA specific to amaranth. PPR proteins are essential for mitochondrial and chloroplast biogenesis, as well as plastid-to-nucleus communication, processes fundamental for retrograde signaling between the plastid and nucleus (RSBPN). In this study, we identified the target of Ahyp-miR0005 and its association with the PPR protein family across *Amaranthus hypochondriacus*, *Arabidopsis thaliana*, *Nicotiana tabacum*, and *Solanum lycopersicum*. Cleavage inhibition by Ahyp-miR0005 was predicted, and the distribution of miRNA binding sites per target gene was analyzed, including their localization within coding PPR domains. Among the main Ahyp-miR0005 target genes, we identified *GUN-1*, *ABO5*, and *MORF1*. Interaction network analysis revealed that different target genes are co-expressed in response to the same stimulus. Gene expression profiling with the Arabidopsis eFP Browser revealed substantial transcriptional changes in predicted targets under six abiotic stress conditions. We further show that abiotic stresses alter the expression of Ahyp-miR0005 amaranth target genes. We anticipate that the expression of Ahyp-miR0005 in non-amaranth plants could replicate the reorganization and coordination of gene expression through RSBPN, thereby improving plant tolerance to various abiotic stresses.

## 1. Introduction

RNA editing plays a pivotal role in regulating gene expression and facilitating plant adaptation to diverse environmental conditions [1]. Under stress, gene modulation is orchestrated through nucleus–plastid interactions, where retrograde signaling from plastids to the nucleus (RSBPN) modulates both organellar genomic expression (OGE) and plastid gene expression (PGE), two fundamental processes for stress response in plants [2].

The pentatricopeptide repeat (PPR) protein family plays a central role in multiple RNA editing events. PPR proteins are encoded in the nucleus, translated in the cytoplasm, and transported to plastids, where they mediate various stages of mitochondrial (mtRNA) and chloroplastic mRNA (ChlRNA) processing [3]. Their functions include RNA cleavage, stabilization, editing, and translation [4], thereby impacting gene expression, mRNA maturation, and the translation of organellar-encoded proteins [5]. Additionally, PPR proteins help maintain mRNA stability in both chloroplasts and mitochondria [6] and contribute to plant responses to environmental stress [7]. As an abundant and evolutionarily conserved gene family, PPR genes are believed to have emerged early in eukaryotic evolution [8]. Their prevalence is evident across species: 493 PPR genes have been identified in *Arabidopsis thaliana* [9], 430 in *Amaranthus hypochondriacus* v2.1 [10], 490 in Oryza sativa Kitaake v3.1 [11], and 473 in *Solanum lycopersicum* ITAG4.0 [12].

Several studies in plants carrying mutations in PPR proteins have linked the absence or alteration of specific genes with key biological processes. These include responses to abscisic acid (ABA) [13,14,15,16,17,18,19], sugar metabolism [13,20], and the biosynthesis of mevalonate and methylerythritol phosphate pathways [21]. Additional functions affected by PPR gene mutations include thermotolerance [22,23], defense against fungal pathogens [13], seed dormancy [24], and ethylene signaling [20]. Furthermore, PPR-related processes have been implicated in responses to cold, reactive oxygen species (ROS), salinity, drought, and tetrapyrrole biosynthesis [14,19,25,26,27].

MicroRNAs (miRNAs) are regulators that control gene expression at the post-transcriptional level [28]. The regulation of PPR family genes by miRNAs could modulate the activity of this gene family, potentially mimicking the effects of PPR gene mutations. Although a few miRNAs have been reported to target PPR transcripts [29,30,31], there is no evidence of coordinated regulation of multiple PPR targets by a single miRNA, a mechanism that could have significant implications for plant physiology or stress response. In *Arabidopsis thaliana*, miR161 and miR400 regulate PPR genes involved in developmental processes [29]. In *Populus trichocarpa*, miR474b, miR475a/b, and miR476a regulate 36 PPR genes, related to cold stress response [30]. Similarly, miR1425, specific to *Oryza sativa*, targets seven PPR transcripts [31]. In *Brassica campestris* ssp. *chinensis*, overexpression of miR158 reduces the expression of bra027656, a PPR protein required for pollen germination [32]. Furthermore, in *Medicago truncatula*, miR1507 and miR2118a/b/c have been shown to target members of the PPR gene family [33].

Some phasiRNAs derived from PPR genes may act in trans by targeting other PPR transcripts [34]. The Mp PPR_66 transcript adopts a stem-loop structure that is capable of generating Mpo-miR11692.1, enabling autoregulation in cis [35]. This suggests a feedback mechanism that governs its expression, like the Ath-miR168/At AGO1 and Ath-miR162/At DCL1 loops [29]. In *Amaranthus hypochondriacus*, Martínez Núñez et al. [36] identified 38 PPR genes potentially regulated by Ahyp-miR0005, a specific miRNA, highlighting a post-transcriptional regulatory layer with physiological relevance. Similarly, in rice, 49 miRNAs were predicted to target 54 PPR genes, with 3 (LOC_Os03g17634, LOC_Os07g40820, LOC_Os04g51350) being experimentally validated as miRNA targets [37]. Additionally, in soybean, the PPR gene Glyma09G256600 is simultaneously targeted by gma-miR1508c and gma-miR4413a, demonstrating a negative regulatory effect under both normal and high-temperature conditions [38].

PPR protein-encoding transcripts were identified as targets of an amaranth-specific miRNA, Ahyp-miR0005 [36]. We hypothesize that analyzing Ahyp-miR0005 and its target genes will help identify the regulation of genes encoding PPR proteins in various plant species under abiotic stress conditions, thereby enabling the selection of key PPRs for adaptation to environmental stress and stress tolerance. Understanding which PPR transcripts are collectively regulated by Ahyp-miR0005 across different species is essential for uncovering its impact on key cellular processes in plants. This study aims to identify conserved Ahyp-miR0005-PPR regulatory networks in *Amaranthus hypochondriacus*, *Solanum lycopersicum*, *Nicotiana tabacum*, and *Arabidopsis thaliana*. We began by analyzing the expression profile of PPR genes under abiotic stress conditions in *Arabidopsis thaliana*. Subsequently, we identified conserved PPR genes targeted by Ahyp-miR0005 conserved in *Solanum lycopersicum*, *Nicotiana tabacum*, and *Amaranthus hypochondriacus* and examined their expression patterns in response to drought stress.

## 2. Results

### 2.1. Identification of miRNA Ahyp-miR0005 Target Transcripts

**Figure 1:**
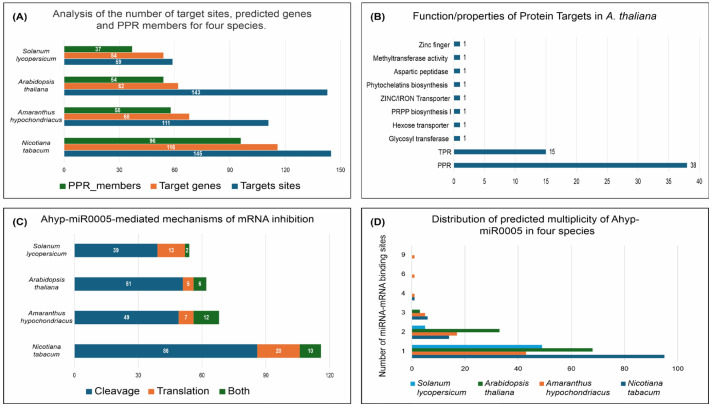
Distribution of miRNA Ahyp-miR0005 target transcripts and their association with the PPR family in four plant species. (**A**) Comparison of target gene number predictions by psRNATarget for four plant species and the number of PPR member genes. (**B**) Annotation of protein targets annotated by Phytozome in *A. thaliana*. (**C**) Analysis of the mechanism of Ahyp-miR0005-mediated inhibition of mRNAs. (**D**) Multiplicity of miRNA-mRNA binding sites in target genes of four plant species.

To identify target genes of Ahyp-miR0005, we employed a strategy based on the approach reported [39]. The analysis of miRNA–target interactions across different plant species revealed a notable disparity between the number of predicted binding sites and the number of target genes. Although the total number of target sites was consistently higher, these sites corresponded to a smaller set of genes, indicating a multiplicity of binding events per gene. For instance, *Arabidopsis thaliana* exhibited 143 target sites distributed across 62 genes, while *Amaranthus hypochondriacus* showed 111 sites associated with 68 genes. Similarly, *Nicotiana tabacum* presented 145 sites linked to 116 genes, and *Solanum lycopersicum* had 59 sites corresponding to 54 genes (Table S2). These findings highlight the potential for individual miRNAs to regulate multiple regions within a single gene or across several genes, reflecting the complexity and versatility of post-transcriptional regulation in plants (Figure 1A). The number of target genes was further reduced when considering that Ahyp-miR0005 can bind to different isoforms of the same transcript (Figure 1A). The annotations available in Phytozome for *Arabidopsis thaliana* allowed us to associate a functional description with 61 proteins derived from the 143 transcripts identified (Figure 1B). Of these, 86.88% (53 of 61) belong to the pentatricopeptide/tetratricopeptide repeat (PPR/TPR) protein family, representing 10.75% of the total PPR family members in Arabidopsis, which has 493 annotated PPR/TPR proteins (Figure 1B). This analysis suggests a strong preference for miRNA in regulating genes within the PPR family, indicating a potentially significant functional role in the post-transcriptional regulation of this family.

### 2.2. Prediction of Cleavage Inhibition and Multi-Site Regulation Analysis of miRNA Ahyp-miR0005

The prediction of Ahyp-miR0005-mediated gene regulation revealed varying patterns of cleavage and translational inhibition across the analyzed plant species. In *Amaranthus hypochondriacus*, 49 target genes (72.06%) were predicted to undergo cleavage, while 12 genes were subject to both cleavage and translational inhibition. In *Arabidopsis thaliana*, 51 genes (82.26%) were predicted to be cleaved, and six genes exhibited both regulatory mechanisms. Similarly, in *Nicotiana tabacum*, 86 genes (74.14%) were predicted to be cleaved, and 10 genes were predicted to be regulated by both mechanisms. In *Solanum lycopersicum*, 39 genes (72.22%) were predicted to be cleaved, and 2 genes to be regulated by both cleavage and inhibition (Figure 1C). Among the identified target genes, occurrences of multiple binding sites were found in 25 genes (38%) in amaranth, 36 genes (27%) in Arabidopsis, 21 genes (20%) in tobacco, and 5 genes (8%) in tomato (Table S3). In tomato, target gene sequences had up to two binding sites; in Arabidopsis, up to three; in tobacco, up to four; and in amaranth, genes with up to four, six, and even nine miRNA binding sites were identified (Figure 1D).

**Figure 2:**
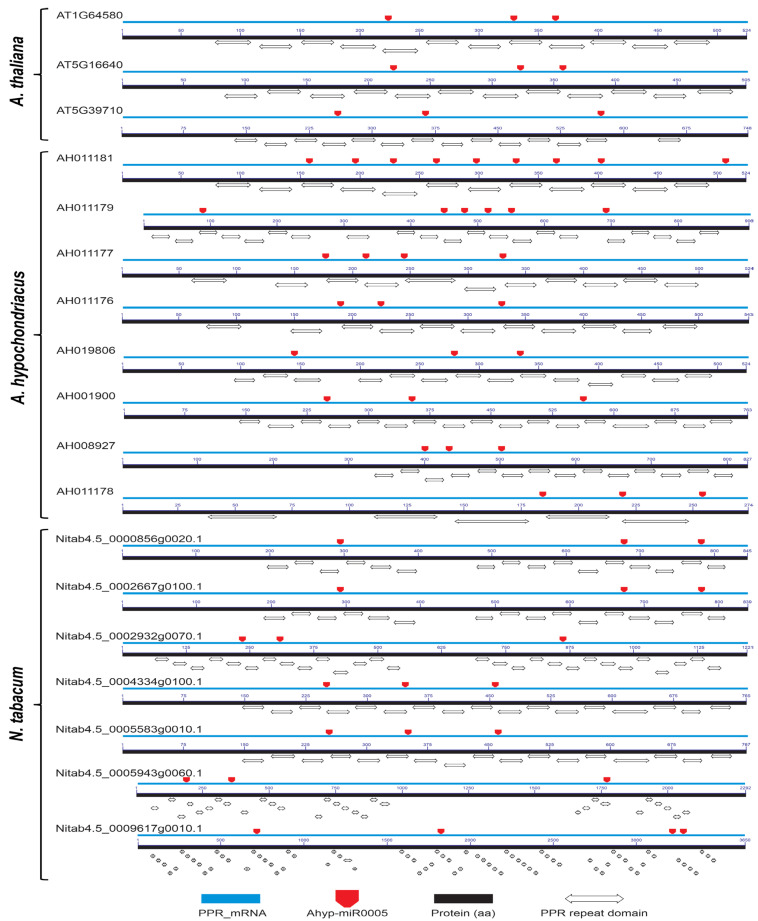
Localization of miRNA-mRNA binding sites within coding PPR domains in amaranth, Arabidopsis, and tobacco. The mRNA is depicted as a blue line, with miRNA binding sites indicated by red arrows. Additionally, the encoded peptide sequence is represented as a black line, on which the conserved domains present in each transcript are highlighted.

The location of these binding sites was determined in genes with at least three sites (Figure 2). In Arabidopsis and tobacco, all binding sites are located within coding regions of PPR domains (Table S3, Figure 2). In *Amaranthus hypochondriacus*, 93.93% (31 of 33) of the miRNA-mRNA binding sites were located within PPR-like coding domains (Figure 2). For the AH011181-RA transcript, eight of the nine binding sites were located within regions encoding PPR domains, and for AH008927-RA, two of the three binding sites were also located in regions encoding PPR domains. These results evidence a high level of redundancy and specificity of the miRNA Ahyp-miR0005 toward PPR domain-coding regions, suggesting targeted and possibly functionally relevant regulation of this family of proteins.

### 2.3. Functional Analysis of Ahyp-miR0005 Target Genes

Since a Gene Ontology annotation of the amaranth genome is not available, we identified the *Arabidopsis thaliana* homologs of the amaranth target genes identified by Ahyp-miR0005. Multiple homologs were found for some genes: 16 for AH018654, 3 each for AH017636 and AH022444, and 2 each for AH012270 and AH014716 (Table S4). GO analysis was performed on the 65 Arabidopsis homologous genes (Figure S1). These Arabidopsis homologs are associated with processes related to mitochondrial RNA modification, mRNA processing, and transmembrane transport, indicating a functional involvement in mitochondrial processes (Figure S1).

An interaction network for Arabidopsis and networks for tomato and amaranth using Arabidopsis homologs were generated with GENEMANIA (Figure S2A), illustrating connections with members of the BASS family, which are involved in transporting monocarboxylic acids across membranes. The co-expression data in GENEMANIA [40] indicated that a considerable percentage of these PPR genes are associated with diverse physiological conditions: 34 genes (52.3%) with cold response, 27 genes (41.5%) with MPK6, 28 genes (43%) with salinity, 15 genes (23%) with plasticity, and 18 genes (27.7%) with ethylene (Figure S2B, Table S5). A prominent case is the AT5G39980 (PDM3—Pigment-Defective Mutant3) gene, which is expressed in six different conditions, suggesting a central regulatory role in the interaction with Ahyp-miR0005. Likewise, co-expression was observed for AT4G20090 (PPR_327), AT5G39710 (PPR_407), and AT1G09900 (PPR_028) with AT1G09820 (PPR_027), AT3G18970 (PPR_243), and AT3G26540 (PPR_256). This distribution suggests a broad functional involvement in the response to different environmental stimuli, which is reflected in the high interconnectivity observed in the co-expression network (Figure S2, Table S5).

### 2.4. Distribution and Phylogenetic Analysis of Ahyp-miR0005 Target Genes

**Figure 3:**
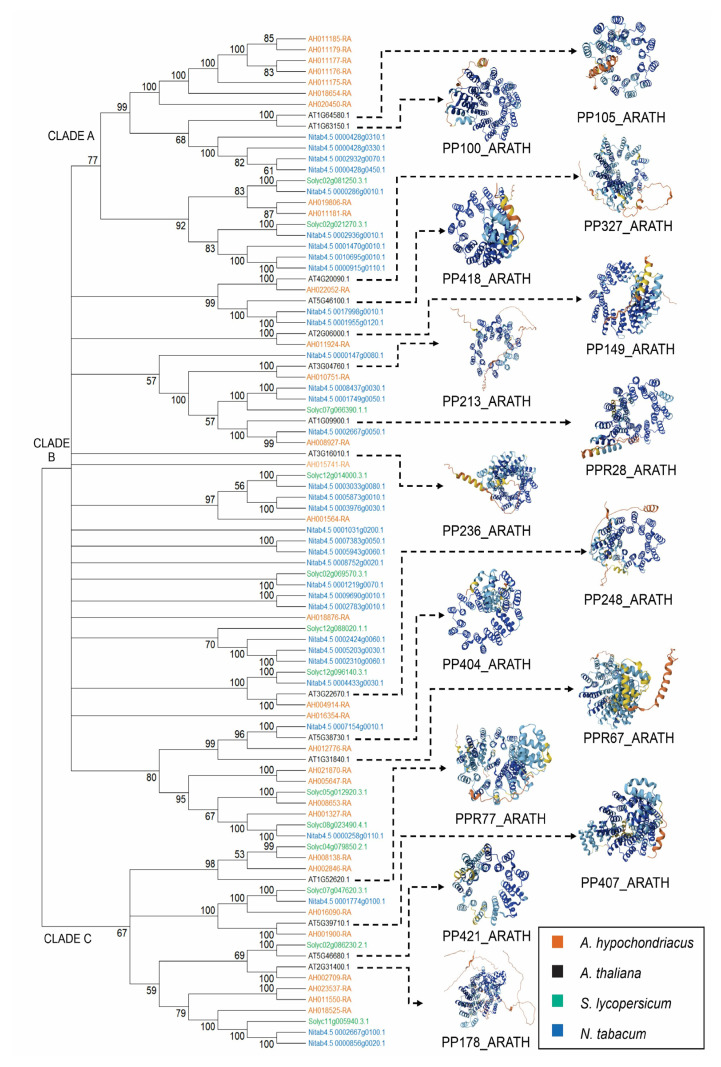
Phylogenetic relationships among predicted Ahyp-miR0005 target genes across different plant species. The subclade orange, corresponding to amaranth, exhibits tight clustering with genetic distances ≤ 0.02 substitutions per site. Transcripts in black (Arabidopsis) appear in mixed clades with bootstrap values ≥ 70. Sequences from *Nicotiana tabacum* (blue) are interspersed with those of *Solanum lycopersicum* (green), also supported by bootstrap values ≥ 70.

To investigate the evolutionary relationships and genomic distribution of the predicted targets of Ahyp-miR0005, a phylogenetic analysis was performed. A total of 106 Ahyp-miR0005 miRNA target genes were identified in the four species analyzed: *Nicotiana tabacum* (39 genes), *Solanum lycopersicum* (13 genes), *Arabidopsis thaliana* (18 genes), and *Amaranthus hypochondriacus* (36 genes). Gene distance estimates between transcripts (Table S6), for target genes where the distance could not be calculated, were discarded. This resulted in 93 transcripts that were aligned and used to construct a phylogenetic tree (Table S6). The phylogenetic tree revealed three main clades, indicating gene family diversification (Figure 3). Clade A, at the top of the tree, groups 22 transcripts with a bootstrap support of 77, divided into two highly supported subclades. Clade B, in the middle section, includes 52 transcripts and shows multiple polytomies that are resolved into short subclades. Clade C, at the bottom, comprises 19 transcripts with an overall bootstrap support of 67. This clade splits into three distinct clusters, with support values of 98 and 100, and a third cluster with a support value of 59 (Figure 3). Overall, 14.3% of the internal nodes have bootstrap values below 70, mostly found in the middle and lower regions of the tree, while the remaining divisions are strongly supported.

The top subclade, corresponding to amaranth, showed tight clustering with genetic distances ≤ 0.02 substitutions per site, suggesting a recent common ancestor or the presence of highly conserved paralogous genes. Transcripts corresponding to Arabidopsis are found in mixed clades with bootstrap values of ≥70, suggesting the presence of orthologous transcripts or conserved regions between species that share similar functions. Conversely, most *Nicotiana tabacum* sequences are intermingled with those of *Solanum lycopersicum*, also with bootstrap values of ≥70, reflecting their close evolutionary relationship within the Solanaceae family. These findings provide insight into the evolutionary dynamics and potential functional conservation among Ahyp-miR0005 target genes, offering a valuable foundation for future functional validation studies.

To compare amaranth genes with those of other species, conserved sequences were identified in the genomes of tomato, Arabidopsis, and tobacco. Twenty-three Ahyp-miR0005 target genes of amaranth were also PPR targets in other species (Table 1). This suggests that they may be involved in RNA-regulated processes and functional conservation across species.

### 2.5. Gene Expression of Ahyp-miR0005 Targets in Arabidopsis Thaliana Under Abiotic Stress

**Figure 4:**
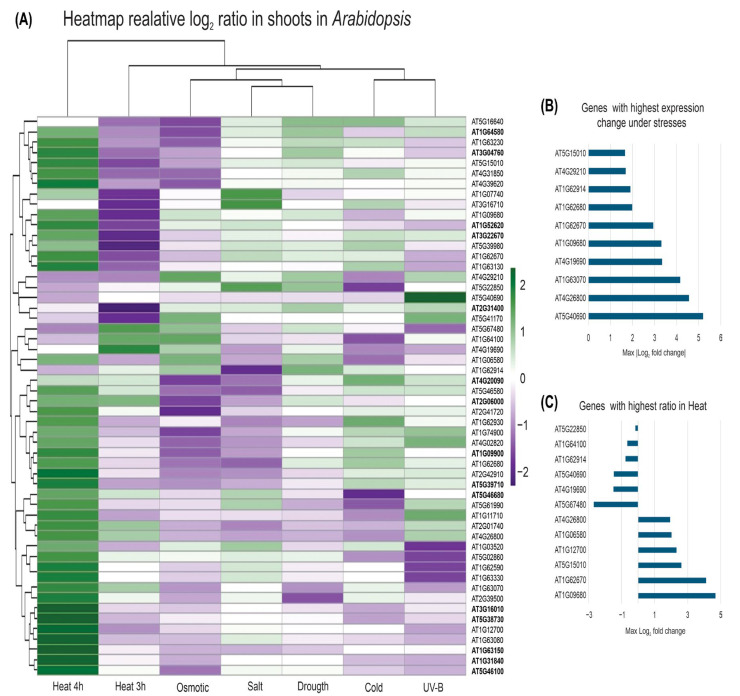
Gene expression under abiotic stress conditions using Arabidopsis eFP Browser. (**A**) Heat map of gene expression under abiotic stress conditions. Relative expression change values (log_2_FC) of target genes predicted by psRNATarget (Expect ≤ 3.5) in response to six stress conditions are shown: cold (4 °C), osmotic (300 mM mannitol), salinity (150 mM NaCl), drought (15 min hot air), UV-B (15 min fluorescence), and heat (38 °C for 3 h and 4 h with recovery at 25 °C). (**B**) Bar graph of target genes with highest absolute log_2_FC change in expression under abiotic stress. (**C**) Differential gene expression variation between 3 h and 4 h of thermal treatment.

We then recovered published Arabidopsis gene expression data to identify Ahyp-miR0005 homolog target genes that respond to six different abiotic stress conditions (Table S7). A heat map was created by analyzing the predicted PPR target genes of miR0005 under abiotic stress (Figure 4A). UV-B radiation strongly upregulated the expression of AT5G40690, showing a log_2_FC value of +5.2, equivalent to an approximately 36-fold increase. Heat treatment caused significant increases in the expression levels of AT4G26800 and AT1G63070. After 3 h of heat exposure, AT4G19690 was upregulated, while AT1G09680 and AT1G62670 were significantly repressed. Under salinity stress, the expression of AT1G62914 decreased. In response to osmotic stress, AT4G19690 was induced, and AT1G62670 was downregulated. Cold stress led to the upregulation of AT1G63070 and the repression of AT4G26800. Finally, during drought treatment, AT4G19690 was upregulated, while AT4G26800 showed decreased expression. Several genes exhibited significant changes in expression (Table S8, Figure 4B). The most contrasting genes in their expression were also identified (Table S8, Figure 4C), which may help us select candidate genes for further study.

Overall, the most extreme transcriptional changes were observed under heat and UV-B conditions, while the other conditions elicited more moderate but consistent changes. A striking finding was the dynamic response observed in the heat recovery condition (heat 4 h), where AT1G09680 and AT1G62670, which were strongly repressed at 3 h (log_2_FC ≈ −3), showed moderate induction at 4 h, with an increase of greater than 4 log_2_ units. This transition suggests rapid transcriptional turnover, which could be key to understanding early signaling mechanisms in response to heat stress and regulation during the recovery phase.

### 2.6. Gene Expression Analysis of Ahyp-miR0005 Targets Under Abiotic Stress in Amaranth

**Figure 5:**
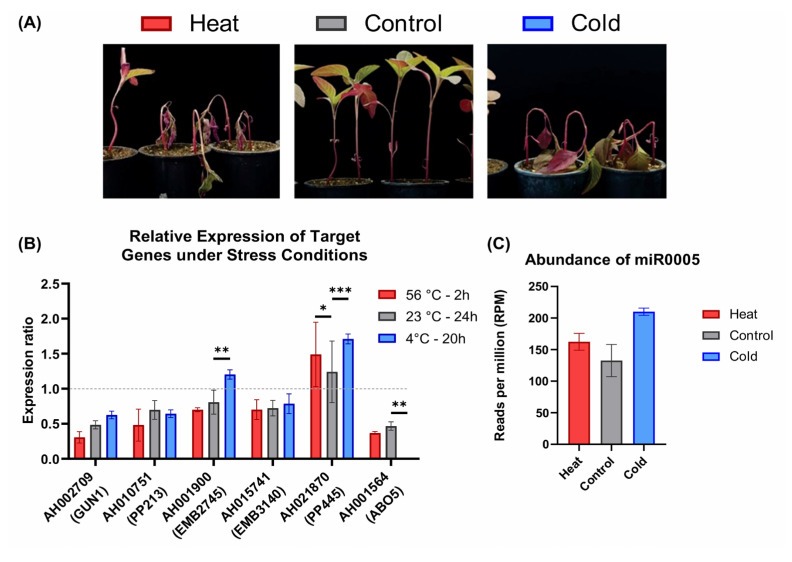
Phenotypic and genetic response of amaranth to heat and cold stress. (**A**) Effect of heat (2 h—56 °C) and cold (20 h—4 °C) stress on amaranth plants. (**B**) Expression profile of PPRs under cold and heat stress conditions in amaranth. Statistical analysis: * *p* < 0.05; ** *p* < 0.01; *** *p* < 0.001 (2-way ANOVA) with α = 0.05 and *n* = 3. (**C**) Expression profile of PPRs under cold and heat stress conditions in amaranth.

Here, we examined the expression of six genes of Ahyp-miR0005 target genes in amaranth, whose homologs in Arabidopsis were identified as stress-responsive. The genes were analyzed in amaranth plants under cold and heat stress conditions (Figure 5A). The differential expression was obtained for each gene (Figure 5B). Exposure to cold results in an upregulation of *GUN1*, *EMB2745*, and *PP445*, while *ABO5* expression decreased. Under heat stress, the expression of *GUN1* and *ABO5* decreased, whereas *PP445* was upregulated (Figure 5B). The relatively low standard deviation values compared to the means across the three biological replicates suggest a high level of reproducibility in the expression data. To support the relationship between miR0005 and PPR genes, we incorporated the expression data of miR0005 in response to cold and heat based on extensive sequencing data from sRNAs (Figure 5C) [36].

## 3. Discussion

### 3.1. Ahyp-miR0005 Targets PPR Genes Across Different Plant Species

The identification of putative target transcripts for Ahyp-miR0005 across four plant species reveals both conservation and divergence in target genes, likely reflecting species-specific regulatory patterns. *Solanum lycopersicum* exhibited the lowest number of predicted targets (59 genes). In contrast, *Amaranthus hypochondriacus* presented 111 predicted targets. A high proportion of the predicted Ahyp-miR0005 targets in both *Amaranthus hypochondriacus* and *Arabidopsis thaliana* were PPR genes, with 54 and 55 targets, respectively, representing 12.55% and 11.15% of their total annotated PPR genes. This enrichment is notable, considering the functional specialization of PPR proteins in RNA-related processes, particularly those involved in mitochondrial and chloroplast mRNA metabolism [41]. The variation in the number of target genes across species could be influenced by genome size, as well as differences in 3′-UTR length and sequence motifs. These observations underscore the importance of considering transcript isoform diversity and the presence of multiple binding sites when interpreting miRNA target predictions, particularly in cross-species contexts [42].

The recurrence of PPR targets across these phylogenetically distant species indicates that Ahyp-miR0005 could represent an interesting miRNA for modulating organellar gene expression. Additional functional studies are needed to verify the regulatory relationship between Ahyp-miR0005 and its predicted targets and to determine whether this miRNA modulates conserved core pathways or drives species-specific adaptations to environmental stress.

### 3.2. Ahyp-miR0005-PPR Interactions with Multiple Binding Sites as Combinatorial MECHANISMS of Gene Regulation

The regulatory sensitivity of miRNA–target interactions often depends on both the number and spatial organization of miRNA binding sites [29,43]. In *Amaranthus hypochondriacus,* analysis revealed up to nine Ahyp-miR0005 binding sites within individual PPR transcripts (Figure 1D and Figure 2), a level of multiplicity that may reinforce post-transcriptional silencing through synergistic effects on mRNA destabilization or translational repression [44,45]. The spatial arrangement of these sites may further contribute to regulatory precision and redundancy, allowing dynamic responses to fluctuations in miRNA concentration [46]. Consistently, the presence of multiple Ahyp-miR0005 binding sites within PPR genes suggests a combinatorial mechanism of regulation, supporting a more refined and robust control of gene expression [47,48,49,50].

PPR transcripts with multiple binding sites may also act as competitive endogenous RNAs (ceRNAs), functioning as miRNA sponges that sequester Ahyp-miR0005 molecules. Similar roles have been described for long non-coding RNAs, pseudogenes, circular RNAs, and protein-coding transcripts, all of which can modulate miRNA availability under specific physiological contexts [48,51]. This potential sponge-like behavior introduces a new layer of regulatory complexity to PPRs, thereby expanding the spectrum of miRNA-mediated control in *Amaranthus hypochondriacus*.

### 3.3. Ahyp-miR0005 Could Regulate Conserved PPR Genes Between Species

PPR proteins are recognized as ancient and pivotal players in organelle biogenesis and adaptive genome evolution [52]; the targeted modulation by miRNAs may reflect a conserved regulatory role in plant resilience. Considering the broad evolutionary conservation of PPR genes across plant species and the regulatory role of Ahyp-miR0005 in amaranthus, we investigated whether it could potentially target PPR genes in other plant species. The phylogenetic analysis of Ahyp-miR0005 target genes across *Nicotiana tabacum*, *Solanum lycopersicum*, *Arabidopsis thaliana*, and *Amaranthus hypochondriacus* provides important insights into the evolutionary pathway and genomic organization of these miRNA target genes (Figure 3). Clustering into three main clades, supported by high bootstrap values in most nodes, reflects both lineage-specific expansions and the preservation of conserved transcripts across species. Notably, the tight grouping of amaranth sequences with low genetic distances suggests either a recent duplication event or conservation of paralogs potentially under stabilizing selection. Additionally, 23 targets of Ahyp-miR0005 in amaranth were also PPR targets in other species (Table 1).

Overall, the evolutionary relationships in this analysis indicate that Ahyp-miR0005 may regulate both conserved and species-adapted regulatory frameworks. These findings provide a critical groundwork for functional assays aimed at determining how miRNA–target interactions have diversified or been maintained across plant lineages and highlight the importance of integrating phylogenetic and molecular data to elucidate the role of Ahyp-miR0005-PPR genes.

### 3.4. Modulation of PPRs and Its Implications in the Abiotic Stress Response

Co-expression profiling of Arabidopsis homologs further supported their physiological relevance, with 52.3% being associated with the cold response, followed by enrichment for salinity stress (43%), MPK6-mediated signaling (41.5%), ethylene response (27.7%), and developmental plasticity (23%) (Figure S2). The largest gene interaction network correlated with cold stress acclimation [53], while a secondary module aligned with root salinity response, echoing earlier findings [54]. These findings emphasize the complexity of miRNA-mediated regulation and its potential impact on stress response mechanisms in plants. Further experimental validation will be essential to clarify the specific roles of these genes in environmental adaptation.

The modulation of PPR genes by Ahyp-miR0005 suggests a complex and finely tuned regulatory landscape involved in plant responses to abiotic stress. Gene expression analysis of predicted targets showed transcriptional changes in response to cold, salt, and osmotic stress in Arabidopsis (Figure 4). In contrast, heat stress caused the most different pattern, suggesting unique regulatory mechanisms or the activation of specific PPR groups. Genes such as AT1G62350 and AT3G01580 were upregulated across all stress conditions despite lacking direct functional validation, while AT1G11290, linked to plastid mRNA editing via DYW motifs [55], and AT2G17033, which encodes an SMR domain-containing protein similar to *GUN1* [56], were downregulated under heat and cold stress. A subset of ten genes displayed simultaneous upregulation under both heat and cold, highlighting potential temperature-responsive candidates. Among these, AT1G63080, AT1G31920, and AT1G63150 have previously been associated with abiotic stress, particularly in the context of mitochondrial electron transport inhibition, an event known to activate MPK3 and MPK6, key players in ROS signaling [57]. The expression dynamics of PPR genes under abiotic conditions underscore their central role in RNA metabolism and organellar homeostasis. Their enrichment as predicted targets of Ahyp-miR0005 in both amaranth and Arabidopsis suggests functional conservation of miR0005-mediated responses.

Meanwhile, *ABO5* is implicated in ABA and cold stress responses. Mutants of abo5 exhibit elevated proline levels and upregulation of genes encoding mitochondrial proteins, which may contribute to enhanced cold tolerance in amaranth. Here, the ABO5 gene was decreased in cold stress in amaranth, and miR0005 increased (Figure 5B,C) [14]. The ABA-responsive genes *ABO5* and *ABO6* are involved in mitochondrial mRNA splicing and maturation, suggesting that ABA-mediated alternative splicing is a key mechanism for coordinating developmental and stress responses in plants [58]. *GUN1* and *EMB2745* increased in amaranth; *GUN1* has similar behavior in Arabidopsis and amaranth (Figure 5B). This interaction likely represents a mechanism for organellar adaptation and stress resilience in plants.

### 3.5. Functional Implications of Ahyp-miR0005-PPR Genes and Their Significance in Organelle Biogenesis

**Figure 6:**
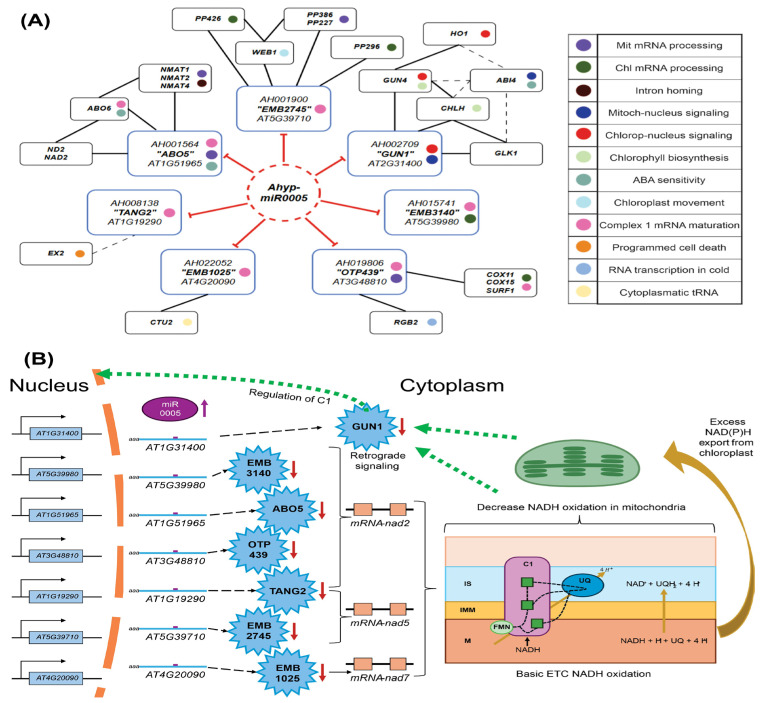
Interactions of Ahyp-miR0005 with orthologous genes and their implication in organelle biogenesis. (**A**) Interaction diagram centered on the Ahyp-miR0005 (red circle), which regulates conserved target genes (blue squares) among four species. These target genes are functionally connected to other associated genes (black squares), through experimentally demonstrated interactions (solid black lines) or inferred by text mining (dotted lines). To the left of each node is a color code indicating the biological function of the gene, including mitochondrial or chloroplast mRNA processing, retrograde signaling, chlorophyll biosynthesis, and ABA sensitivity, among others. (**B**) PPR protein-mediated regulation of mitochondrial Complex I and its impact on retrograde signaling. PPR proteins (stars) show a decrease in response to an increase in Ahyp-miR0005. Six target genes participate in the processing of nad2, and five in that of nad7, both essential genes for the formation of NADH dehydrogenase (Complex I or C1) of the electron transport chain (ETC). The primary oxidation of NADH occurs in this complex. Excess NAD(P)H is exported to the nucleus (yellow arrows), and both mitochondria and chloroplasts send retrograde signals to the nucleus (green arrow) to regulate C1 expression.

GO analysis indicated that the predicted Ahyp-miR0005 target genes are primarily involved in mitochondrial RNA modification, mRNA processing, and transmembrane transport, supporting a role in organelle regulation (Figure S1). This reinforces the established function of PPR genes in maintaining mitochondrial gene expression and metabolic integrity under diverse physiological conditions. The Ahyp-miR0005–PPR gene network, which includes conserved target genes across species, suggests that this miRNA regulates proteins involved in electron transport (Complex I), mitochondrial and plastid dynamics, protoporphyrin synthesis, hormonal responses (ABA, ethylene), and morphogenesis (Figure 6). Genes associated with processes such as chloroplast movement (*WEB1*), chlorophyll biosynthesis (*GUN4*, *CHLH*), programmed cell death (*EX2*), and cold-responsive transcription (*RGB2*) are linked to Ahyp-miR0005–PPR regulation, indicating a complex regulatory network controlled by Ahyp-miR0005 (Figure 6A).

*GUN1* plays a pivotal role in retrograde signaling, aligning nuclear gene expression with plastid status and facilitating plastid biogenesis (Figure 6A) [59]. Several Ahyp-miR0005 target genes, including *ABO5*, *TANG2*, *OTP439*, *EMB2745*, *EMB1025*, and *EMB3140*, are associated with Complex 1 (NADH dehydrogenase), a key component of the mitochondrial electron transport chain (Figure 6B). Dysfunction of this complex leads to NADH accumulation and an altered NADH/NAD^+^ ratio, which negatively affects core metabolic pathways, including the Krebs cycle, β-oxidation of fatty acids in glyoxysomes, and gluconeogenesis [60]. This suggests that Ahyp-miR0005 may influence organelle biogenesis through gene regulation and modulate retrograde signaling by targeting mitochondrial Complex I via PPR-mediated mechanisms (Figure 6). This includes PPR genes regulated by Ahyp-miR0005 that are involved in the maturation and processing of mitochondrial NAD transcripts (e.g., NAD2, NAD5, NAD7). Mutations in genes such as *ABO5*, *EMB3140*, *TANG2*, and *EMB1025* impair Complex I activity, reducing NADH oxidation, an effect like that of miR0005-mediated regulation. The resulting accumulation of NAD(P)H may be transported to the chloroplast, altering its redox state and triggering retrograde signaling to the nucleus, where GUN1 integrates signals to reprogram nuclear gene expression. This connection implies a potential regulatory influence of Ahyp-miR0005 on electron transfer activity during NADH oxidation. It will be important to clarify the effects of Ahyp-miR0005 on mitochondrial remodeling and adaptive stress responses.

## 4. Materials and Methods

### 4.1. Prediction of Ahyp-miR0005 Target Genes and Their Action Mechanisms

Target prediction was carried out using the psRNATarget online platform (A Plant Small RNA Target Analysis Server, 2017 Update; [61]), applying default parameters, with the exception of setting the expectation value to 3.5, against the *A. hypochondriacus* cDNA library (version 2.1; [10]). A similar process was applied to additional species using their respective cDNA libraries: *Arabidopsis thaliana* (version 11; [9]), *Solanum lycopersicum* (ITAG4.0; [12]), and *Nicotiana tabacum* (version 4.5; [62]). The slicing site of miRNA target sequences was defined at nucleotide positions 10 and 11. A mismatch or bulge within this region inhibits protein translation without inducing cleavage. Therefore, miRNA–target pairs with perfect complementarity in this region were considered potential cleavage candidates. After each target prediction analysis, the number of target genes belonging to the PPR family was determined using gene annotations available in Phytozome https://phytozome-next.jgi.doe.gov/ (accessed on 27 February 2025).

### 4.2. Analysis of Conserved Domains in High-Multiplicity Target Genes

To explore the functional relevance of highly targeted genes, coding sequences (CDSs) with three or more predicted miRNA binding sites (high multiplicity) were subjected to conserved domain analysis. The analysis was conducted using the Conserved Domain Database (CDD) from the U.S. National Library of Medicine, version 3.21, which comprises 62,456 position-specific scoring matrices (PSSMs). An E-value threshold of 0.01 was applied to identify statistically significant domain matches https://www.ncbi.nlm.nih.gov/Structure/cdd/wrpsb.cgi (accessed on 27 February 2025).

CDSs were downloaded from Phytozome from Arabidopsis and amaranth in FASTA format, and the tobacco CDSs were downloaded from SolGENOMICS https://solgenomics.net/ (accessed on 3 February 2025) [63]; the CDSs were then aligned with their respective full-length mRNA transcripts. miRNA binding sites were mapped onto these sequences based on predictions from psRNATarget. This integration enabled the identification of binding sites located within coding regions, particularly those overlapping with conserved PPR domains.

### 4.3. Gene Ontology (GO) Enrichment Analysis and Interaction of Target Genes

To identify biological categories that are over- or under-represented among the Ahyp-miR0005 target genes, Gene Ontology (GO) enrichment analysis was conducted using AgriGO2 (http://systemsbiology.cau.edu.cn/agriGOv2/; accessed on 29 August 2025 [64]). For the Ahyp-miR0005 target genes of Arabidopsis and the homologous genes predicted by Phytozome for tomato and amaranth, enrichment was evaluated through Singular Enrichment Analysis (SEA), and significantly over-represented GO terms—within the biological process, cellular component, and molecular function categories—were identified using a false discovery rate (FDR)-adjusted *p*-value threshold of 0.05.

### 4.4. Co-Expression and Functional Interaction Analysis of Homologous Target Genes in Arabidopsis Thaliana

Gene interaction analysis was performed using the GeneMANIA platform https://genemania.org/search/arabidopsis-thaliana/ (accessed on 7 April 2025), focusing on co-expression networks associated with specific conditions: cold, circadian cycle, salinity, ethylene, plasticity, and MPK6 (Mitogen-Activated Protein Kinase 6). The data obtained were visualized in Cytoscape_v3.10.3, where a gene interaction network was constructed. Each group of co-expressed genes under a specific condition was assigned a distinct color label, facilitating the visual interpretation of the co-expression patterns and their potential functional relevance in various physiological and environmental contexts.

### 4.5. Gene Expression Analysis Under Abiotic Stress Conditions Using Arabidopsis eFP Browser

Target genes predicted with an expectation value ≤3.5 were selected for expression analysis in the Arabidopsis eFP Browser https://bar.utoronto.ca/eplant/ (accessed on 14 April 2025). Expression data reported by Kilian et al. [65] were obtained. Data for cold (4 °C), osmotic stress (300 mM mannitol), salinity (150 mM NaCl), drought (15 min exposure to hot air), UV-B radiation (15 min fluorescent light, followed by 25 °C), and heat (38 °C) were used [65]. Relative expression change values (log_2_FC) at 3 h of treatment were downloaded for the six conditions mentioned, as well as for an additional heat condition with recovery (38 °C for 3 h followed by 25 °C) [65]. With this data, a heat map was generated using R version 4.5.0, along with a graph showing the highest absolute values of gene expression change. In addition, the expression delta between the 3 and 4 h heat conditions was calculated, highlighting the genes with the highest variations in this condition.

### 4.6. Phylogenetic Analysis of Ahyp-miR0005 Target Genes in Four Species

PPR targets predicted by psRNATarget were selected to determine their conservation among plant species. A selection of Ahyp-miR0005 miRNA target genes was performed by applying an expectation threshold ≤ 2.5 in the four species analyzed. Sequences corresponding to these transcripts were downloaded from Sol Genomics Network https://solgenomics.net/ (accessed on 21 April 2025) [63] for the genus tobacco and from Phytozome https://phytozome-next.jgi.doe.gov/ (accessed on 21 April 2025) for the remaining species.

The sequences were aligned in MEGA12 [66] using the ClustalW (DNA) algorithm with default parameters. To estimate the genetic distance between transcripts, the two-parameter Kimura substitution model (K2P) with 1000 bootstrap replicates was applied, considering both transitions and transversions. This analysis allowed us to identify and exclude transcripts with high variability or errors in distance estimation.

Subsequently, a phylogenetic tree was constructed using the Neighbor-Joining method with 1000 bootstrap replicates, and evolutionary distances were calculated using the p-distance method. Each species was assigned to a color code for easy visualization. Finally, the predicted protein structure of the homologous transcript in Arabidopsis thaliana was downloaded from the UniProt database https://www.uniprot.org/ (accessed on 25 April 2025) [67].

### 4.7. Plant Growth Conditions

Seeds of the “Gabriela” variety of *A. hypochondriacus* L. were provided by MsC. Roberto Bernal-Muñoz from the Instituto Tecnológico del Altiplano de Tlaxcala, México. The growth conditions were indicated by Martínez [36]. Briefly, seeds were sterilized with 10% sodium hypochlorite for 5 min, followed by a treatment with 50% ethanol for 1 min. After each immersion, the seeds were washed three times for 3 min with sterile water. Seeds were sown in 1″ × 1″ × 2.5″ polystyrene trays containing sterile Magic Grower (Green Monkey—Magic Grower Rojo, Puebla, Mexico). Plants were grown in a greenhouse under natural daylight conditions, with a photo period of 14 h light/10 h darkness, and temperatures ranging from 21 to 39 °C. They were watered every third day until they developed the sixth true leaf.

Six amaranth plants in the first vegetative stage (sixth true leaf, [36]) of *A hypochondriacus* variety Gabriela were subjected to stress treatment until half of the treated population showed signs of damage. The cold treatment was carried out at 4 °C for 20 h, while the heat treatment occurred at 56 °C for 2 h. A photographic record was taken at the end of the experiment, and the tissue was preserved at −80 °C until analysis. Each experiment was conducted in triplicate.

### 4.8. Total RNA Extraction

RNA was extracted using the TRIzol method, following a modified protocol from tissue preserved at −80 °C [68]. Briefly, 50 mg of tissue was macerated in 100 µL of 8 M LiCl on ice. Then, 1 mL of TRIzol was added, and the mixture was homogenized for 15 s using vortexing. Next, 200 µL of chloroform was added, and the mixture was homogenized again. It was centrifuged at 12,000 rpm for 15 min at 4 °C, and then 400 µL of the supernatant was recovered into a new tube. Then, 800 µL of chloroform was added, and the mixture was homogenized. It was centrifuged at 12,000 rpm for 15 min at 4 °C, and 400 µL of the supernatant was transferred into a new tube. Then, 1 mL of isopropanol was added to precipitate the RNA for 2 h at −20 °C. After centrifugation at 12,000 rpm for 15 min at 4 °C, the pellet was washed with 1 mL of 75% ethanol. After centrifugation at 7500 rpm for 10 min at 4 °C, the ethanol was decanted to dry the RNA pellet, and the pellet was then resuspended in 25 µL of DEPC-treated water. The quality and integrity of total RNA were checked on a 1.5% Agarose gel and quantified with a NAS-99 NuDrop spectrophotometer (ATCGene, Piscataway, NJ, USA).

### 4.9. Quantification of Target Genes

The primers were specifically designed for each PPR gene and synthesized by T4Oligo (ADN ARTIFICIAL S DE RL, Irapuato, Mexico). Polyadenylation of the RNA was performed as a template for cDNA synthesis. This involved the addition of 1 µL of poly(A) polymerase 10× reaction buffer (NaCl 250 mM, Tris-HCl 50 mM, MgCl_2_ 10 mM, pH 7.9), 1 µL of ATP 10 mM, 0.1 µL of ribonuclease inhibitor (Sigma Aldrich, Cat. No. R1158, Saint Louis, MO, USA), and 0.16 µL of poly(A) polymerase (5000 U/mL, New England Biolab, Cat. No. M0203S, Ipswich, MA, USA) to 500 ng of RNA, gauged to 10 µL. The mixture was incubated for 40 min at 37 °C and inactivated for 10 min at 65 °C.

To synthesize cDNA, 2 µL of the polyadenylated RNA was mixed with 1 µL of 0.25 µM stem-loop primer RT and 0.5 µL of 10 nM dNTP, made up to 8 µL. The mixture was heated for 5 min at 70 °C and then cooled in an ice bath for 5 min. Then, 2 µL of 5X RT reaction buffer, 0.1 µL of ribonuclease inhibitor, and 0.5 µL of OneScript^®^ Plus Reverse Transcriptase (ABM Inc., Cat. No G237, Whittier, CA, USA) were added to the reaction. The cDNA synthesis reactions were performed for 10 min at 25 °C, followed by 50 min at 52 °C, and then 5 min at 85 °C.

Expression of target genes was determined by semiquantitative RT-PCR. The oligonucleotides used for amplifying the target genes are listed in Table S1. The Ahyp_*GAPDH* housekeeping gene was used as a control (Table S1) and reported as a reference gene of amaranth [69]. Endpoint PCR was performed using 1 μL of cDNA as a template (200 ng/μL). Quantification was performed with three technical and three biological replicates of each condition. To estimate the relative variation in expression, the values for each gene were divided by the corresponding value for the constitutive gene, producing the change ratios. Results were compared to determine the expression profile of these PPR genes in amaranth.

## 5. Conclusions

The identification of putative targets for Ahyp-miR0005 showed that miR0005 mainly targets PPR genes across various plant species, emphasizing its potential role in regulating organellar gene expression. Ahyp-miR0005 binds to multiple sites on PPR genes, allowing for combinatorial regulation and possible sponge activity. Several PPR family members, which display different levels of expression under abiotic stress conditions, were identified as predicted targets of Ahyp-miR0005, suggesting that the interaction between Ahyp-miR0005 and PPR genes is important for plant acclimation and stress tolerance. Bioinformatic results indicate a critical miR0005-PPR gene regulatory network, so more research should be conducted on Ahyp-miR0005 to clarify its properties and functions. To determine whether all PPRs are regulated by miR0005 across various stress types or if the response is specific to each stress type. Therefore, conducting studies to elucidate the function of miR0005 will undoubtedly be key to considering its regulation and application. These analyses will be essential to explain the broader physiological effects of Ahyp-miR0005 regulation, particularly in relation to mitochondrial remodeling and adaptive stress responses.

## Figures and Tables

**Table 1:** Conservation of shared Ahyp-miR0005 target genes of amaranth targets among the different species.

Amaranth	Name	Ath_Name UniProt	Arabidopsis	Tomato	Tobacco	Function
AH001327	-	F4I4T7_ARATH	AT1G30290	Solyc08g023490	Nitab4.5_0002071g0050.1 Nitab4.5_0000315g0050.1	-
AH001564	ABO5	PPR76_ARATH	AT1G51965	Solyc03g121110	Nitab4.5_0005085g0030.1 Nitab4.5_0004969g0060.1	Embryogenesis, metabolism
AH001900	EMB2745	PP407_ARATH	AT5G39710	Solyc01g108410	Nitab4.5_0004334g0100.1 Nitab4.5_0005583g0010.1	Essential in embryo
AH002709	GUN1	PP178_ARATH	AT2G31400	Solyc06g009520.3.1	Nitab4.5_0000363g0190.1 Nitab4.5_0007407g0040.1	Stress response
AH002755	-	-	-	Solyc03g114000 Solyc06g071310	Nitab4.5_0001422g0090.1 Nitab4.5_0009569g0010.1 Nitab4.5_0002314g0060.1 Nitab4.5_0004495g0010.1	-
AH002846	-	PP440_ARATH	AT5G61400	Solyc06g069700	Nitab4.5_0003021g0070.1 Nitab4.5_0004286g0010.1	RNA processing
AH004914	-	PP211_ARATH	AT3G04130	Solyc05g014490	Nitab4.5_0001898g0020.1	RNA processing
AH005647	-	PP445_ARATH	AT5G65560	Solyc10g081880	Nitab4.5_0002165g0010.1 Nitab4.5_0005257g0020.1 Nitab4.5_0002364g0030.1 Nitab4.5_0000574g0030.1	RNA processing
AH008138	TANG2	PPR50_ARATH	AT1G19290	Solyc04g079850.2.1	Nitab4.5_0006518g0030.1 Nitab4.5_0000110g0140.1	RNA processing
AH008653	-	PP388_ARATH	AT5G16420	Solyc01g096210	Nitab4.5_0002527g0080.1 Nitab4.5_0002667g0050.1	RNA processing
AH008927	MEE40	PP281_ARATH	AT1G09900	-	Nitab4.5_0002667g0060.1 Nitab4.5_0002667g0050.1	RNA processing
AH010751	-	PP213_ARATH	AT3G04760	-	Nitab4.5_0003780g0130.1	RNA processing
AH011550	ABO8	PP306_ARATH	AT4G11690	-	Nitab4.5_0007891g0010.1 Nitab4.5_0001714g0210.1	Response to abscisic acid
AH011924	-	PP149_ARATH	AT2G06000	Solyc01g104630	Nitab4.5_0009570g0010.1 Nitab4.5_0000249g0340.1	RNA processing
AH012776	-	PP180_ARATH	AT2G32630	Solyc10g084080	Nitab4.5_0005518g0020.1 Nitab4.5_0001297g0100.1	RNA processing
AH015741	PDM3/EMB3140	PP408_ARATH	AT5G39980	Solyc02g087560.1	Nitab4.5_0002265g0130.1 Nitab4.5_0000564g0400.1	-Essential in Embryo -Chloroplast development
AH016090	-	PP338_ARATH	AT4G26680	Solyc07g047620	Nitab4.5_0001774g0100.1 Nitab4.5_0001701g0020.1	RNA processing
AH016354	EMB1444	PPR15_ARATH	AT1G06143	-	Nitab4.5_0000128g0270.1	Essential in embryo
AH018525	-	PP325_ARATH	AT4G19440	Solyc11g005940	Nitab4.5_0000856g0020.1 Nitab4.5_0002667g0100.1	RNA processing
AH018654	RPF1 PPR3 - RFL9/RPF4 RPF6 RPF8 - RFL2 - - RPF2 - - PPR-AC RPF3 -	PPR38_ARATH PP247_ARATH PP100_ARATH PPR94_ARATH PPR99_ARATH PPR37_ARATH PPR98_ARATH PPR36_ARATH PP102_ARATH PPR97_ARATH PPR91_ARATH PPR39_ARATH PP101_ARATH PPR90_ARATH PPR96_ARATH PP103_ARATH	AT1G12700 AT3G22470 AT1G63150 AT1G62910 AT1G63130 AT1G12620 AT1G63080 AT1G12300 AT1G63400 AT1G63070 AT1G62670 AT1G12775 AT1G63330 AT1G62590 AT1G62930 AT1G64100	Solyc06g007740.1 Solyc06g007850 Solyc04g080120.1 Solyc06g007300 Solyc05g009253.1 Solyc06g005220.2	-	-RNA processing factor -mRNA modification
AH019806	OTP439	PP270_ARATH	AT3G48810	Solyc01g058205.1	Nitab4.5_0000225g0060.1 Nitab4.5_0004303g0030.1	RNA processing
AH021870	-	PP445_ARATH	AT5G65560	Solyc07g047820	Nitab4.5_0000441g0020.1 Nitab4.5_0011940g0010.1	RNA processing
AH022052	EMB1025	PP327_ARATH	AT4G20090	Solyc02g081860	Nitab4.5_0000844g0300.1 Nitab4.5_0010610g0010.1	Essential in embryo
AH023537	-	PP156_ARATH	AT2G16880	Solyc01g111470.3.1	Nitab4.5_0000061g0180.1	RNA processing

Ahy-miR005 target genes in amaranth were examined in other species; conserved target genes of amaranth with tomato, tobacco, and Arabidopsis were identified in Phytozome; conserved target genes with tobacco were identified in NCBI.

## Data Availability

The data used to generate and support the findings of this study are publicly available or are contained within this article and its Supplementary Material.

## Supplementary Materials

The following supporting information can be downloaded at https://www.mdpi.com/article/10.3390/plants14172757/s1, Figure S1. GO enrichment analysis of Ahyp-miR0005 target genes in *Arabidopsis*, tomato, and amaranth. Figure S2. Cytoscape co-expression interaction network. Blue (cold), green (circadian cycle), red (salinity), orange (ethylene), purple (plasticity), and yellow (MPK6). Figure S3. Electrophoresis of PPRs under cold and heat stress conditions in amaranth. Table S1. Oligonucleotides used for Ahyp-miR0005 targets. Table S2. Target genes predicted by psRNATarget for selected species. Table S3. Number of binding sites with multiplicity by species. Table S4. Orthologs of amaranth in other species. Table S5. Cytoscape stress co-expression from GENEMANIA. Table S6. Mega distances calculated for PPR targets of Ahyp_miR005. Table S7. Expression profile of PPR transcripts under abiotic stress conditions for Arabidopsis. Gene expression under stress conditions using the Arabidopsis eFP Browser. Cold (4 °C), osmotic (300 mM mannitol), salinity (150 mM NaCl), drought (15 min hot air), UV-B (15 min fluorescence), and heat (38 °C for three and four hours with recovery at 25 °C). Table S8. Changes in EFP values with the highest expression change under stress and heat stress.

## Abbreviations

The following abbreviations are used in this manuscript:

miRNAs	MicroRNAs
PPR	Pentatricopeptide repeat
RSBPN	Retrograde signaling between the plastid and nucleus
PPR/TPR	Pentatricopeptide/tetratricopeptide repeat
ROS	Reactive oxygen species
GUN 1	GENOMES UNCOUPLED 1
ABO5	ABA overly-sensitive 5
MORF1	Multiple organellar RNA editing factor
BASS	Bile acid sodium symporter
GO	Gene ontology
ceRNAs	Competitive endogenous RNAs
CDS	Coding sequences
CDD	Conserved Domain Database
PSSMs	Position-specific scoring matrices
MPK6	Mitogen-Activated Protein Kinase 6
ABA	Abscisic acid
SEA	Singular Enrichment Analysis
