# Supplementary figures and images for: Bioinformatics Analysis Reveals PPR Genes Modulation by Ahyp-miR0005 Under Abiotic Stress Across Diverse Plant Species
Source: Plants (Basel). 2025 Sep 3;14(17):2757. doi: 10.3390/plants14172757 (PMC12430068; doi:10.3390/plants14172757)

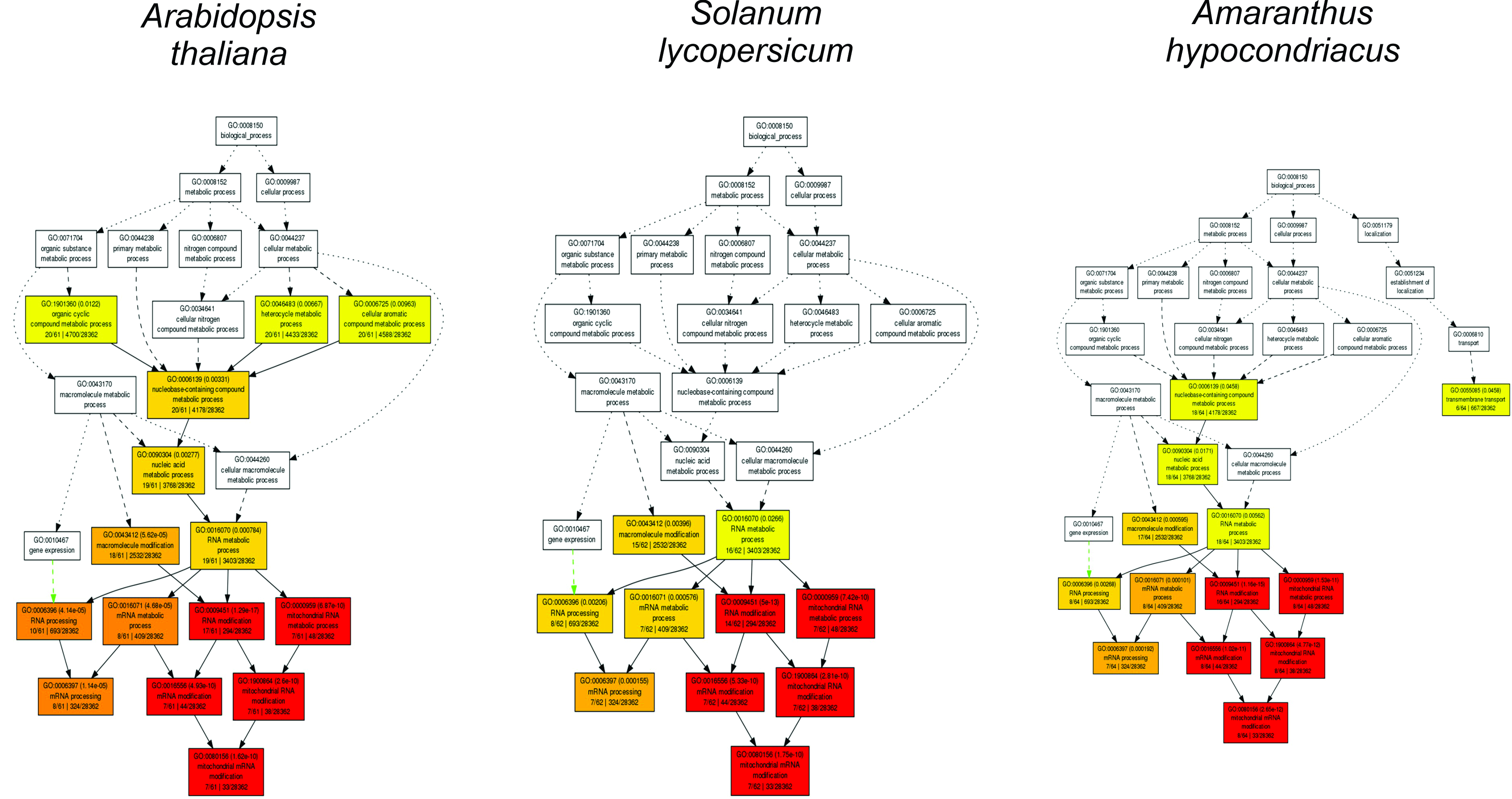

Supplement: Supplementary file 1 [file plants-14-02757-s001.zip › Figura S1 300 DPI.jpg]

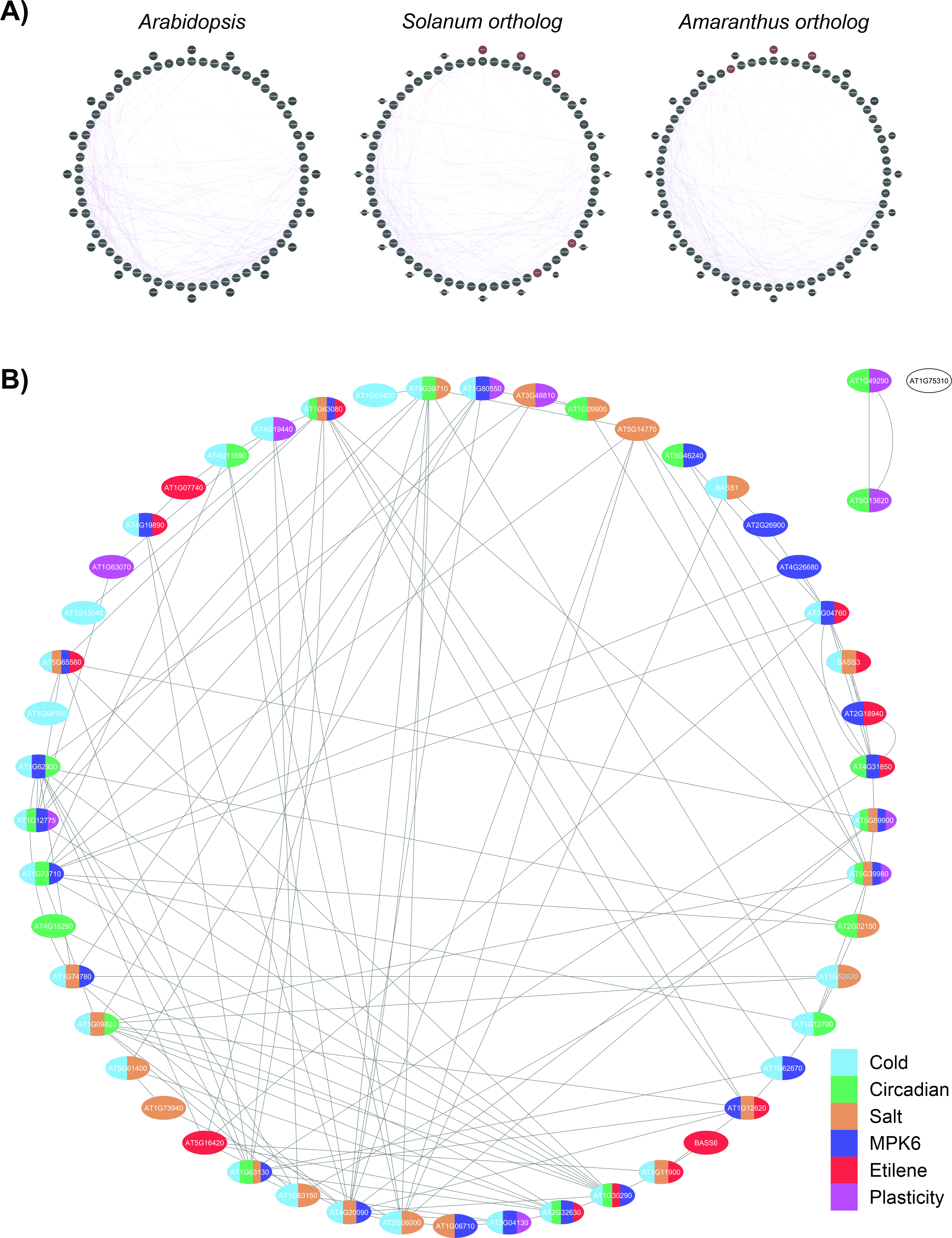

Supplement: Supplementary file 1 [file plants-14-02757-s001.zip › Figura S2 300 DPI.jpg]

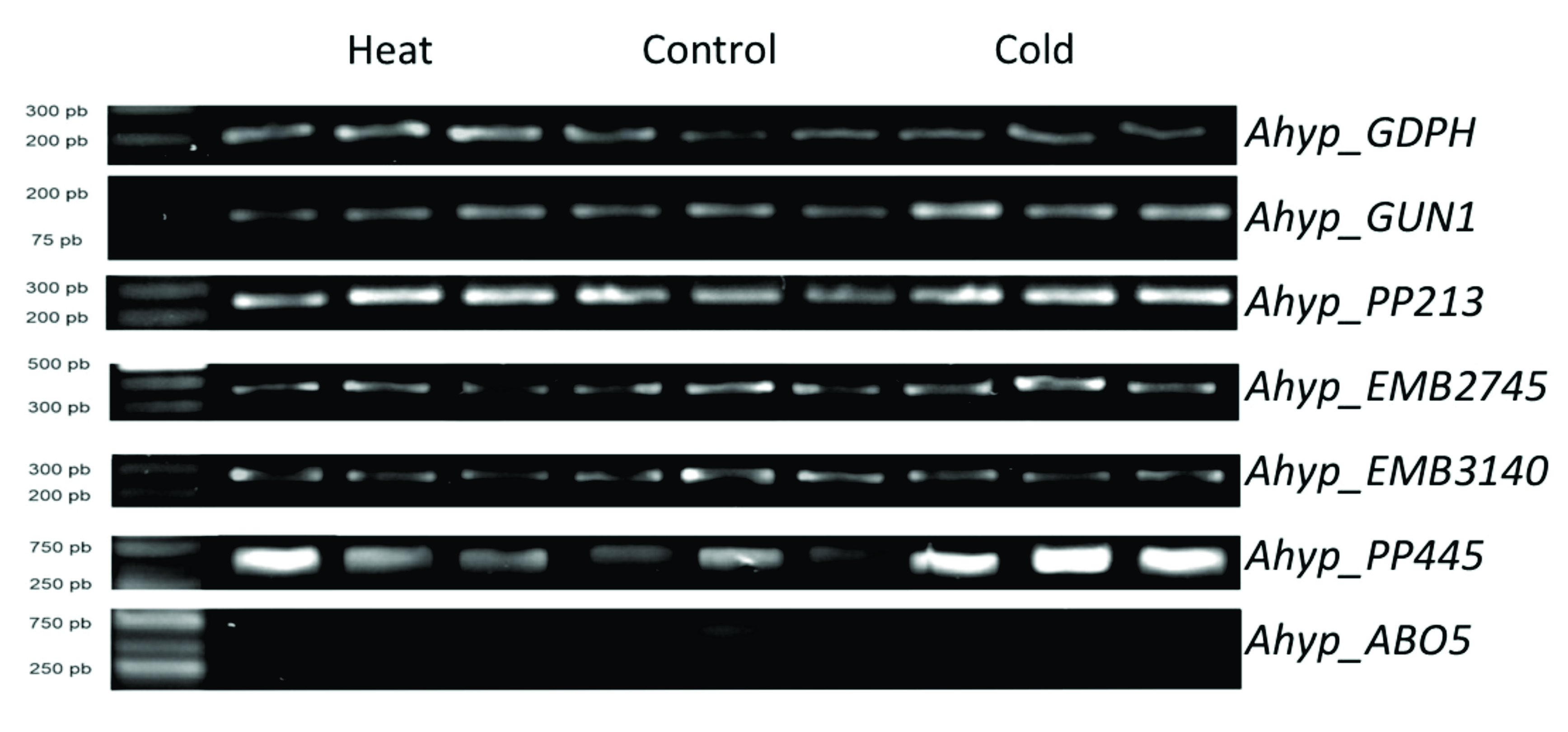

Supplement: Supplementary file 1 [file plants-14-02757-s001.zip › Figura S3 300 DPI.jpg]
